# Robust, small-scale cultivation platform for *Streptomyces coelicolor*

**DOI:** 10.1186/1475-2859-11-9

**Published:** 2012-01-17

**Authors:** Sujata Vijay Sohoni, Prashant Madhusudan Bapat, Anna Eliasson Lantz

**Affiliations:** 1 Center for Microbial Biotechnology, Department of Systems Biology, Technical University of Denmark, Building 223, DK-2800 Kgs Lyngby, Denmark; 2 Present address: Novozymes A/S, Krogshoejvej 36, 2880 Bagsvaerd, Denmark; 3 Present address: Novozymes A/S, Hallas Alle 1, 4400 Kalundborg, Denmark

## Abstract

**Background:**

For fermentation process and strain improvement, where one wants to screen a large number of conditions and strains, robust and scalable high-throughput cultivation systems are crucial. Often, the time lag between bench-scale cultivations to production largely depends on approximate estimation of scalable physiological traits. Microtiter plate (MTP) based screening platforms have lately become an attractive alternative to shake flasks mainly because of the ease of automation. However, there are very few reports on applications for filamentous organisms; as well as efforts towards systematic validation of physiological behavior compared to larger scale are sparse. Moreover, available small-scale screening approaches are typically constrained by evaluating only an end point snapshot of phenotypes.

**Results:**

To address these issues, we devised a robust, small-scale cultivation platform in the form of MTPs (24-square deepwell) for the filamentous bacterium *Streptomyces coelicolor *and compared its performance to that of shake flasks and bench-scale reactors. We observed that re-designing of medium and inoculum preparation recipes resulted in improved reproducibility. Process turnaround time was significantly reduced due to the reduction in number of unit operations from inoculum to cultivation. The incorporation of glass beads (ø 3 mm) in MTPs not only improved the process performance in terms of improved oxygen transfer improving secondary metabolite production, but also helped to transform morphology from pellet to disperse, resulting in enhanced reproducibility. Addition of MOPS into the medium resulted in pH maintenance above 6.50, a crucial parameter towards reproducibility. Moreover, the entire trajectory of the process was analyzed for compatibility with bench-scale reactors. The MTP cultivations were found to behave similar to bench-scale in terms of growth rate, productivity and substrate uptake rate and so was the onset of antibiotic synthesis. Shake flask cultivations however, showed discrepancy with respect to morphology and had considerably reduced volumetric production rates of antibiotics.

**Conclusion:**

We observed good agreement of the physiological data obtained in the developed MTP platform with bench-scale. Hence, the described MTP-based screening platform has a high potential for investigation of secondary metabolite biosynthesis in *Streptomycetes *and other filamentous bacteria and the use may significantly reduce the workload and costs.

## Background

*Streptomycetes *produce numerous primary as well as secondary metabolites. Currently more than half of the antibiotics in clinical use are produced by *Streptomyces *species [1,2]. Traditionally *Streptomyces *strain improvement programs have been dominated by random mutagenesis, followed by screening and selection in controlled environments for a desired phenotype [3]. Today, with the advent of industrial systems biology [4], this traditional approach is complemented with targeted genetic strategies to increase yield, titer, productivity, and/or robustness [5,6]. Similarly, medium optimization strategies have also played a significant role in improving the productivity in antibiotic cultivation processes. The fate of these high-throughput approaches in capturing the desired phenotype lies in the successful scaled down [7] version of the production process i.e. to keep the physiological traits constant when the scale is changed. Moreover, such low volume, high-throughput cultivation platforms (HTPs) can also be used in other process optimization projects [8]. Small scale optimization of bioprocesses using microtiter plates (MTPs) has generated considerable interest in the research community over the last years [9-14]. However, efforts towards low volume cultivations for filamentous organism have been sparse [15,16]. Major challenges in development of a HTP for filamentous organisms are attributed to (i) unit operations involved from spore plate to cultivation, which make the process low-throughput as well as add variability and ii) morphology, that adds noise in the cultivation process resulting in lower reproducibility, thereby a less robust performance. Furthermore, pH management might be difficult in MTPs, however, this is a general issue not only valid for filamentous organisms. The efforts toward validating MTP in terms of scale comparison (bench-scale reactor versus MTP) and reproducibility at trajectory scale are sparse. Hence, MTP is typically applied as an end point snapshot tool for screening, whereas less attention has been paid towards mapping the entire trajectory of the underlying phenotype. This could be critical as microorganisms may have more than one optimum for deriving the same phenotype [17].

In this communication we report for the first time a systematic scale down approach for *S. coelicolor *cultivations that includes evaluation of kinetic physiological parameters and not only end point values. An optimization of the MTP cultivation was undertaken aiming at reducing process time as well as to enhance reproducibility and obtain a performance similar to that of bench-scale reactors. The developed platform was validated for reproducibility and parameters were compared with the ones obtained from the bioreactor cultivations.

## Materials and Methods

Solvents were HPLC grade and all other chemicals were analytical grade and purchased from Sigma -Aldrich (Steinheim, Germany) unless otherwise stated. Water (MQ) was purified using a Milli-Q-system (Millipore, Bradford, MA).

24-square deepwell microtiter plates and sandwich covers that contained special filters to minimize water evaporation during cultivations were obtained from Enzyscreen BV (Leiden, Netherlands) and used for all MTP cultivations in this study.

### Strain

**Figure 1:**
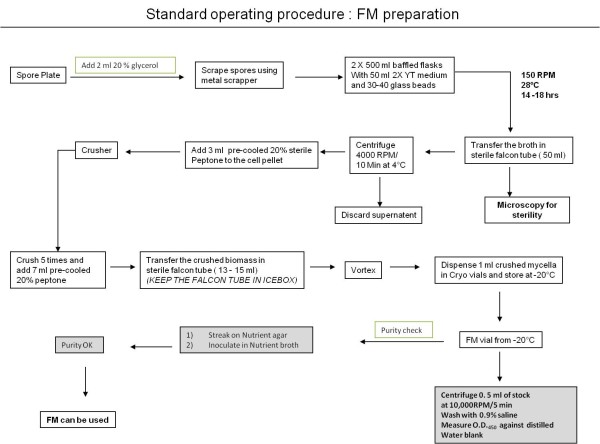
**Preparation of frozen mycelia**. Frozen mycelium was prepared from a spore plate of *S. coelicolor *incubated for 8 days. Boxes with grey filling represent quality control steps.

*Streptomyces coelicolor *A3(2) was a kind gift from Mervyn Bibb, John Innes Centre, Norwich, UK. The cultures of *S. coelicolor *were cultivated from frozen mycelia (FM) vials as described below. **Preparation of frozen mycelia **The protocol described by Borodina *et al. *(2008) for inoculum preparation of *S. coelicolor *was modified as shown in Figure 1. *S. coelicolor *was grown on Soya flour mannitol agar plates until well sporulated. Spores were harvested with 2 ml of 20% (v/v) glycerol and filtered through glass fiber wool to remove mycelial fragments. The resulting spore suspension was then used to inoculate two 500 ml volume baffled shake flasks with 50 ml 2X YT medium and 30-40 glass beads (3 mm diameter) as described by Borodina *et al. *(2008). The shake flasks (SF) were incubated at 28°C and 150 rpm [18]. One of the flasks was used to follow the growth (pH and optical density measurements) by taking duplicate samples every 3 hrs. The other flask, to be used for frozen mycelia (FM) preparation, was harvested when the culture had reached mid exponential phase, typically at pH 8.1. The entire broth was centrifuged at 4,000 × g for 10 min at 4°C; care was taken not to include the beads while transferring the broth to a sterile falcon tube. The supernatant was discarded aseptically and the pellet was resuspended in 3 ml of 20% (w/v) precooled sterile peptone. The falcon tube was kept on ice for five minutes to prevent rise in the temperature during crushing. The mixture was then transferred into a pestle and tube glass homogenizer (in-house fabricated) and crushed gently moving the pestle up and down five times. 7 ml peptone was added to the crushed mycelia. OD_450 nm _of the resulting mycelia stock was measured and recorded (OD_450 nm _at around 25). The mycelia stock solution was dispensed into cryo vials and stored at -20°C.

The purity of FM stock was checked by inoculation in nutrient broth and plating on nutrient agar plates. All the steps described above were carried out in laminar airflow to ensure aseptic conditions.

### Preparation of medium

The defined minimal medium used for bench-scale cultivations was a modification to Evans medium (1970), and was limited in phosphate. The medium was prepared as described by Borodina *et al. *(2008) and contained 3 mM NaH_2_PO_4_, 100 mM NH_4_Cl, 10 mM KCl, 2 mM Na_2_SO4, 2 mM citric acid as chelating agent, 1.25 mM MgCl_2_, 0.25 mM CaCl_2_, as well as the following per liter: 30 g glucose, 5 ml trace elements solution (20 mM FeCl_3_, 10 mM CuCl_2_, 50 mM ZnCl_2_, 10 mM MnCl_2_, 0.02 mM Na_2_MoO_4_, 20 mM CoCl_2_, 10 mM H_3_BO_4_), 1 ml vitamins solution (0.05 g of biotin, 1 g of calcium pantothenate, 1 g of nicotinic acid, 25 g of myo-inositol, 1 g of thiamine-HCl, 1 g of pyridoxine-HCl, 0.2 g of para-aminobenzoic acid/liter) and 100 μl of organic antifoam. The medium used for MTPs and shake flasks was exactly the same as the one for bench-scale cultivations, except that it contained 3-(N-morpholino) propanesulfonic acid (MOPS) as buffer and no antifoam. It should be noted that for un-optimized MTPs, 100 mM 2-(*N*-morpholino) ethanesulfonic acid (MES) was used as a buffer instead of MOPS. Medium pH for MTPs and shake flasks was adjusted in the range of 6.80 to 6.90 using sterile NaOH solution (4 M).

### Sterilization of bioreactors, shake flasks and MTPs

Bioreactors were sterilized with the medium at 121°C for 40 minutes. Sterile glucose and vitamins were aseptically added to the sterile bioreactor.

Baffled shake flasks used for cultivations were autoclaved with 30-40 3 mm glass beads at 121°C for 40 minutes. The flasks were retained at 55°C for removal of moisture if any.

Each MTP well was filled with glass beads. The entire assembly was placed in a sterilization pouch (Westfield medicals, UK), heat sealed and autoclaved at 121°C for 35 to 40 minutes. All the MTPs were kept in wrapped condition at 55°C before inoculation to remove moisture if any.

For shake flask and MTP cultivations, the medium was autoclaved separately for 20 minutes.

### Cultivation conditions

Batch cultivations were performed in bioreactors with 1 L working volume (Applikon Biotechnology, Schiedam, Netherlands) at 28°C, pH 6.80-6.90, 500 rpm agitation rate and a 1 vvm aeration rate. Bioreactors were inoculated with 1 ml frozen mycelia stock. The bioreactors were equipped with a cooling condenser to avoid evaporation from the medium. pH was maintained using 2 M NaOH solution. The concentrations of CO_2 _and O_2 _of the off-gas were monitored using an acoustic gas analyzer (1311, Innova Air Tech Instruments A/S. Naerum, Denmark).

For shake flask cultivations, 50 μl frozen mycelia stock was mixed with 50 ml of sterile medium and added to each flask. Shake flasks were incubated at 28°C and 150 rpm. For batch cultivations performed in MTPs, 80 μl of frozen mycelia stock was mixed with 80 ml of sterile re-constituted medium. 3.15 ml of this mixture was dispensed into each well of a sterile MTP. The MTPs were incubated at 28°C and 150 rpm. The shakers used for shake flasks and microtiter plate cultivations were orbital shakers with 25 mm diameter of shaking.

### Sampling

The samples were taken at regular intervals throughout the cultivations for analyses of pH, glucose and antibiotics. For bioreactor cultivations, 13 ml sample was taken at each time point and 10 ml was used for dry cell measurement (DCW). When OD_450 nm _of biomass was above 1, the quantity of broth for each DCW determination was reduced to 5 ml and duplicate measurements were analyzed. The remaining 3 ml sample was preserved for later antibiotic quantification.

For MTPs optical density was used initially for biomass measurements. When OD_450 nm _> 1 samples were taken for dry cell weight (in duplicate). Around 2.8 ml broth from each MTP well was taken and 2.4 ml was used for DCW and the remaining 400 μl sample was preserved for antibiotic quantification. Thorough mixing of the sample was ensured while pipetting.

### Dry cell weight (DCW) measurement

Dry cell weights were measured as described by Borodina *et al. *(2008) [18]. Filtrates were preserved for analysis of extracellular metabolites.

### Actinorhodin and Udecylprodigiosin quantification

To extract actinorhodin (ACT), 1.8 ml of 2 M NaOH was added to 200 μl sample. The mixture was vortexed and centrifuged at 10000 × g for 10 minutes at 4°C and the absorbance of the supernatant measured at 640 nm. For extracting undecylprodigiosin (UDP), 1.8 ml of acidified methanol (pH 1.5) was mixed with 200 μl sample. The entire mixture was kept on shaking overnight at 2°C. The absorbance of the supernatant was measured at 530 nm after centrifugation at 10000 × g for 10 minutes. The antibiotic concentrations were calculated as described in Borodina *et al. *(2008) [18]. All the measurements were done in duplicate.

### Analysis of substrate and extracellular metabolites

The concentrations of glucose and organic acids were analyzed by HPLC using a HPX-87H column (Bio-Rad Laboratories, California, USA). The operating temperature was 60°C. Separation was achieved using 5 mM H_2_SO_4 _at a flow rate of 0.6 ml/min. The HPLC was equipped with a Waters 410 differential refractometer (Millipore) and a tunable absorbance detector set at 210 nm (Waters 486, Millipore, Massachusetts, USA).

### Volumetric particle size distribution measurements

A Mastersizer 2000 (Malvern Instruments Ltd, Worcestershire, UK) unit was used to determine the volumetric cell size distribution in the cultivation broth from MTPs and bench-scale reactors (modified from Petersen *et al. *2008 [19]). This is a light scattering method that infers a volumetric size distribution, reporting the sizes of spheres with equal volume to the particles actually present. In the size distribution data the particles are divided into 31 size classes ranging from 4.75 to 1,610 μm in diameter and for each size class the volume percentage of the particles is reported. The unit consists of two parts, a mixing chamber with agitator and a laser obscuration cell. The mixing chamber was thoroughly washed with MQ water prior to measurements. For the particle size distribution analysis, 25 ml of MQ water was added to the mixing chamber and agitator speed was fixed at 1000 rpm. The broth sample was added until the laser obscuration value reached 12-15%. The measurement was then started and data was recorded. This procedure was repeated three times using the same sample for calculating the average particle size from MTP and bioreactor cultivations, respectively.

## Results

### Optimization of inoculum procedure

To ensure equal distribution of mycelia between wells in the MTPs and also to avoid a time consuming pre-cultivation procedure, we introduced a "single step inoculation" strategy in the form of frozen mycelia, (Figure 1; see also Materials and Methods for more details on the procedure). The mycelia were gently crushed before freezing to minimize pellet formation and to ensure equal distribution among wells when inoculating MTPs. No significant difference was observed in cultivation trajectories when using FM compared to cultivations using the classical method of inoculation (data not shown; [18]), indicating that the crushing of mycelia had no adverse effects on growth and production. This inoculation procedure using FM was applied for all cultivations described below.

### Performance of un-optimized MTP cultivations

The specific growth rate was 0.03 ± 0.008 h^-1 ^across all the MTPs, which was less than the reported specific growth rate of 0.1 h^-1 ^for *S. coelicolor *in bench-scale reactors (Table 1) [18]. Around 15 hrs of lag phase was seen in all the cultivations. In terms of antibiotic production, onset of undecylprodigiosin (UDP) was observed around 25 hrs where as actinorhodin (ACT) started at around 50 hrs. The final titer (at 100 hrs) was 6.4 mg/l and 18.5 mg/l for UDP and ACT respectively, which was a lot lower than normally seen in bench-scale reactors (Table 1).

**Table 1:** Macro parameters in MTP, Shake flask and Bioreactor

	Un-optimized MTP^1^	Optimized MTP^2^	SF^2^	Bioreactor^3^
**μ_max _(h^-1^)**	0.03 ± 0.008	0.11 ± 0.01	0.11 ± 0.01	0.10 ± 0.005
**Final Biomass (g/L)**	2.0 ± 0.13	4.0 ± 0.13	3.7 ± 1.3	4.1 ± 0.1
**UDP (mg/L)**	6.4 ± 1.6	80 ± 4.4	28 ± 15.5	65 ± 3.3
**ACT (mg/L)**	18.5.0 ± 7.5	520 ± 17.0	267 ± 23.6	500 ± 17.7

Biomass, UDP and ACT concentration indicated in the table corresponds to 120 hrs.

^1^Data based on 4 biological replicates each in triplicate. Un-optimized MTPs contained a medium with MES buffer and no glass beads.

^2^Data based on 3 biological replicates each in triplicate. Optimized MTPs and Shake flasks (SF) contained a medium with 100 mM MOPS buffer to maintain constant pH and 3 mm glass beads.

^3^Data based on three cultivations.

### Optimization of MTP cultivations

#### a) Effect of bead size

**Figure 2:**
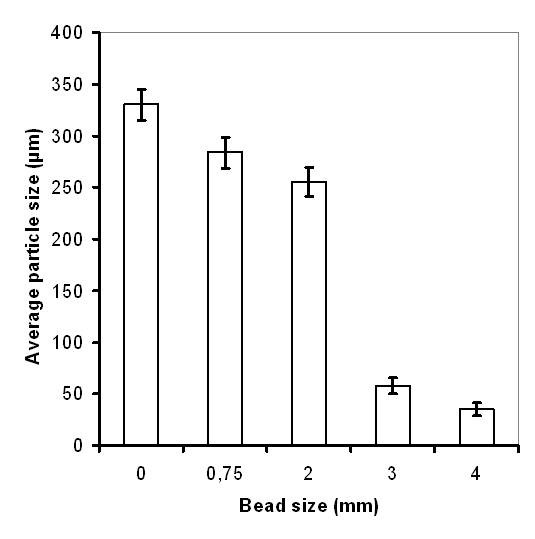
**Effect of bead size on *S. coelicolor *morphology**. Each MTP containing beads of one of the following sizes (no beads, 0.75, 2, 3 and 4 mm) was inoculated with *S. coelicolor*. About 1 ml sample (taken at 48 hrs) from each MTP was loaded into the mixing chamber of Mastersizer 2000 to determine the size distribution. Data shown are mean values from three wells and error bars represent standard deviation.

**Figure 3:**
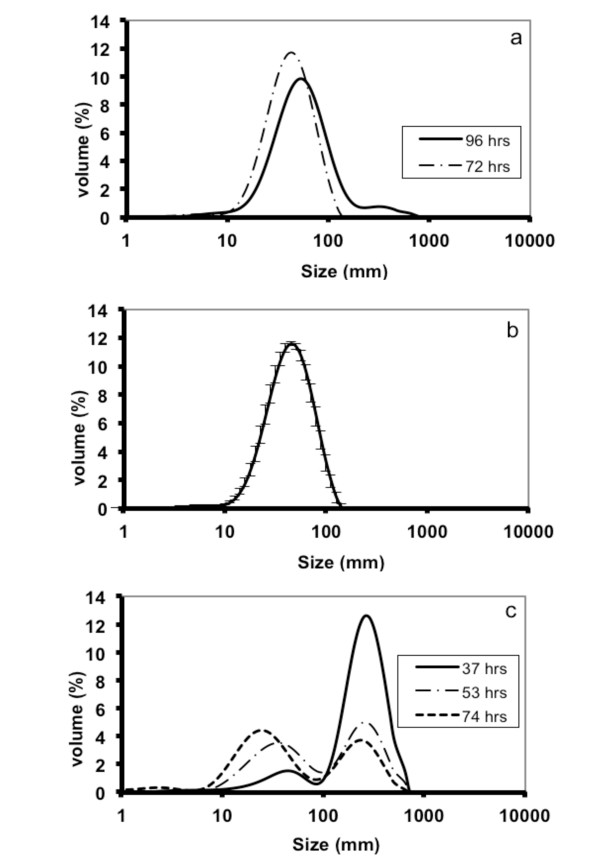
**Morphology of *S. coelicolor *across scales**. a) Particle size distributions at two different time points during growth in MTP. Samples were taken at 72 and 96 hrs from three wells and mean values for each time point is reported. b) Reproducibility across wells in MTPs. Samples was taken from three wells at 72 hrs to estimate mean values. (c) Particle size distribution in bioreactor cultivations. Three samples were taken at each time point (37, 53 and 74 hrs). Mean values are presented in the figure.

In the search for what may cause the poor behavior of the MTP cultivations, large differences in morphology between bench-scale and MTP cultivations were observed. In MTPs, formation of large pellets took place, which was believed to cause well to well variation in terms of growth and antibiotic production and hence, to a large extent be responsible for the poor performance. To avoid pellet formation in MTPs, glass beads were added to each well (six beads per well). Bead sizes in the range 0.75-4.0 mm were evaluated. Samples were taken at regular intervals to monitor growth, antibiotic production as well as volumetric particle size distribution of the biomass. Pellet formation was observed in the MTPs with no beads as well as with 0.75 mm zirconium and 2 mm glass beads. The average size of these pellets was in the range of 255 to 330 μm (Figure 2). Dispersed morphology was observed in the MTPs with glass beads of 3 and 4 mm size. We observed that addition of 3 mm beads not only helped in maintaining the morphology in a narrow size window of 58 ± 8 μm, but also played a significant role towards preventing wall growth. Another major advantage of using glass beads was high reproducibility of mycelial size distribution. The morphology of mycelia not only remained within an acceptable variation limit throughout a cultivation cycle (Figure 3a) but also a high degree of reproducibility was maintained across the wells (Figure 3b). This is very important while screening large numbers of strains or cultivation conditions where morphological reproducibility is one of the key influencing factors. Surprisingly a small peak representing 200 to 400 μm mycelia was observed at 96 hrs (Figure 3a). However, no pellets were detected during microscopic examination and the overall % of this peak is below significance (≤ 0.8% of total biomass volume). For the cultivations with 3 mm beads, ACT and UDP production was also significantly improved, approaching values observed for bench-scale reactors (data not shown). The average particle size was slightly reduced 35 ± 6 μm in the cultivations with 4 mm beads (Figure 2). No ACT production was observed in MTPs with 4 mm beads up to 100 hrs. Hence, we conclude that the use of 3 mm beads is optimal for *S. coelicolor *MTP cultivations. Interestingly we observed multiple size distribution in the cultivations conducted in reactors (Figure 3c). The morphology was more pellet-like (200-400 μm) at the early state of cultivation, i.e. before 40 hrs, whereas a mixed size distribution was observed in the later stages of cultivation where the particles with the same size range as seen for MTPs increased in number. Similar behavior has been reported by Petersen *et al. *2011 [20]. They observed a change in morphology during fed-batch cultivations with *S. coelicolor*, with a smaller size population appearing at around 30-50 h and then increasing in number throughout the cultivation. The shear exerted from agitation might be one of the possible reasons behind the observed morphologies.

#### b) Effect of concentration of MOPS buffer

**Figure 4:**
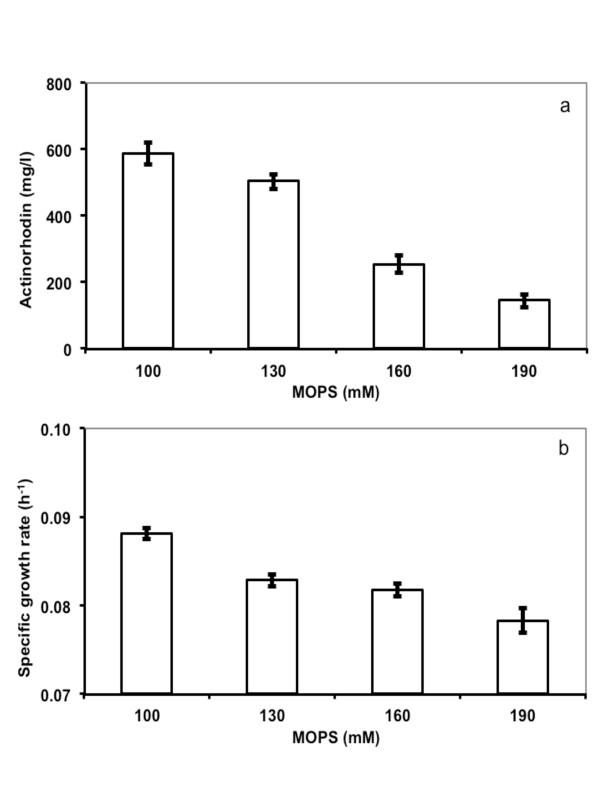
**Effect of MOPS buffer on a) Actinorhodin titer b) Specific growth rate**. Five different MOPS concentrations (50, 100, 130, 160 and 190 mM) were investigated. The concentration of actinorhodin described in the figure is from 144 hrs. Samples from 0 to 48 hrs were used to estimate specific growth rate. Data presented are mean values of three samples. Please note that data from cultivations with 50 mM MOPS was not included in the figure as pH decreased to 5.80 early during cultivation, severely affecting growth and production.

Significant decrease in pH is another factor that may contribute to the poor behavior observed for the un-optimized MTP cultivations. Hence, the MES buffer was replaced with MOPS, which has a higher pK_a _value (7.20 instead of 6.15) with the ability to keep pH above 6.5 and possible inhibitory concentrations of MOPS were evaluated by testing different buffer strengths. *S. coelicolor *FM stock was dispensed in defined medium containing 50, 100, 130, 160 and 190 mM of MOPS, respectively. This pre-inoculated medium was then dispensed in five MTPs (one plate per MOPS concentration) containing six glass beads/well (ø 3 mm). Samples were taken in triplicate at regular intervals to monitor antibiotic production and growth. A significant delay in ACT onset was observed in the batches with higher MOPS concentration (data not shown), which resulted in lower ACT concentrations at the end of the cultivations. ACT concentration was decreased significantly in the batches with 160 and 190 mM MOPS (Figure 4a). Growth rate did also decline with higher MOPS concentrations, although not as drastically as the ACT production (Figure 4b). In cultivations with 50 mM MOPS, pH decreased to 5.8 with poor growth and production as a result, demonstrating that the buffering capacity was not enough at this buffer concentration (data not shown). Consequently, MOPS buffer concentration of 100 mM was optimal for cultivations in terms of onset of ACT production, amounts of ACT produced and growth.

### Validation and performance of optimized MTP

Once the MTP platform was optimized, *S. coelicolor *cultivations were carried out in MTPs, shake flasks and bioreactors (each in triplicate) in order to evaluate the reproducibility of the MTP cultivations and to compare the performance at different scales (3 ml, 50 ml and 1 L, respectively). For MTP an additional fourth cultivation was carried out two weeks after the first three, to further check reproducibility. In all cases phosphate limited medium was employed and FM stock was used as inoculum. For shake flasks and MTPs, the medium was buffered with 100 mM MOPS, whereas 2 M NaOH was used to control the pH at 6.80 in the reactors. Samples were drawn at regular intervals in triplicate. For validating rates of underlying metabolic processes, analysis of physiological traits was performed using standard physiological extracellular parameters.

**Figure 5:**
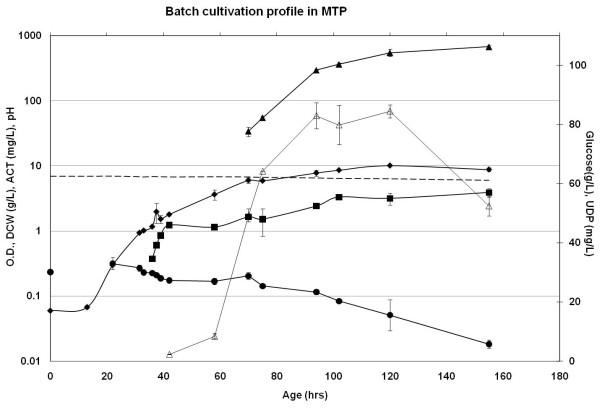
**Batch cultivation profile in the optimized MTP**. *S. coelicolor *was cultivated in MTPs containing 3 ml defined medium with 100 mM MOPS and 3 mm glass beads. Typical time course profiles for the parameters biomass in the form of OD_450 nm _(♦) and DCW (■) g/L, glucose (●) g/L, UDP (Δ) mg/L and ACT (▲) mg/L and pH (--) are shown. OD, DCW, ACT and pH trends are represented on primary axis while, glucose and UDP trends are depicted on secondary axis.

The mean specific growth rate was 0.11 h^-1 ^( ± 0.01) across all the MTPs (Table 1, 2). Around 15 h of lag phase was seen in all the plates (Figure 5). In terms of antibiotic production, onset of UDP was observed around 40 h where as ACT production started at around 60 h. The titer at 120 h was 80 mg/l and 520 mg/l for UDP and ACT respectively. The final biomass concentration was 4.0 g/L. The pH was typically reduced to 6.5 from 6.9 at 100 h of cultivation and further to 6.0 at 155 h. It should be noted that in the cultivations with MES buffer (in un-optimized MTPs), pH dropped to 5.8 immediately after the exponential growth phase at around 40 h. The standard deviation was in the range of ± 5-10% for biomass and antibiotic measurements across all the optimized MTPs. Moreover, physiological parameters were within SD limits of ± 10% (data not shown) for cultivations carried out with different frozen mycelia lots, demonstrating good reproducibility using the optimized MTP procedure.

**Table 2:** Macro parameters in MTPs and in Bioreactors

MacroParameters	MTP1	MTP2	MTP3
μ_max _(h^-1^)	0.10 ± 0.002	0.12 ± 0.01	0.11 ± 0.001
q _UDP _(mg/L/h)	1.9 ± 0.09	1.8 ± 0.13	2.25 ± 0.2
q _ACT _(mg/L/h)	10.9 ± 0.15	8.02 ± 0.6	9.7 ± 1.4
-r_s _(mmoles glu/g DCW/h)	1.8 ± 0.07	1.8 ± 0.07	1.8 ± 0.11
Y_sx _(g/g)	0.32 ± 0.01	0.32 ± 0.01	0.35 ± 0.02

MacroParameters	R1	R2	R3

μ_max _(h^-1^)	0.10 ± 0.01	0.10 ± 0.001	0.11 ± 0.002
q _UDP _(mg/L/h)	1.8 ± 0.02	1.9 ± 0.13	2.1 ± 0.12
q _ACT _(mg/L/h)	11.6	12.4 ± 0.31	10.7 ± 0.001
-r_s _(mmoles glu/g DCW/h)	1.9 ± 0.2	1.82 ± 0.005	1.59 ± 0.13
Y_sx _(g/g)	0.36 ± 0.05	0.31 ± 0.001	0.31 ± 0.02

Triplicate samples were taken from MTPs and Bioreactors at various time points throughout the cultivations to calculate macro parameters indicated in the table.

**Figure 6:**
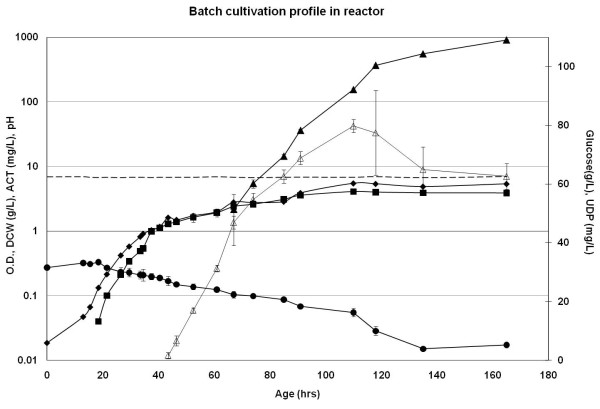
**Batch cultivation profile in the reactor**. *S. coelicolor *was cultivated in a reactor with 1 L defined medium. Typical time course profiles for the parameters biomass in the form of OD_450 nm _(♦) and DCW (■) g/L, glucose (●) g/L, UDP (Δ) mg/L, ACT (▲) mg/L and pH (--) are shown. OD, DCW, ACT and pH trends are represented on primary axis while, glucose and UDP trends are depicted on secondary axis.

In bioreactors a lag phase of around 20 hours was observed (Figure 6.). Time points for onset of UDP and ACT production were the same as those for the MTP cultivations, around 40 hours and 60 hours, respectively. A mean specific growth rate of 0.10 ± 0.01 was observed and titers of UDP and ACT reached 65 mg/L and 500 mg/L, respectively, at 120 hrs. The biomass concentration was 4.1 g/L at the same time point.

Also the kinetic parameters calculated from data obtained in MTPs and bench-scale cultivations were in agreement (Tables 1, 2). Volumetric antibiotic production rates were found to be around 2 mg/L/h and 10 mg/L/h for UDP and ACT respectively, for both MTPs and bioreactors. One could argue that the ACT production was somewhat lower in MTPs as compared to reactors however, the difference was very small. Specific glucose consumption rates (-r_s_) were in the range of 1.6 to 1.9 mmoles.glc/g.dcw/h. Biomass yields were estimated to be around 0.33 (g.dcw/g.glucose) across the scales. The overall standard deviation was below ± 10% for all the parameters.

In the overall comparison of the physiological parameters, MTP and bioreactor cultivations showed good agreement. However, shake flask data differed quite a lot (Table 1). Considerably lower antibiotic production was observed and the reproducibility was a lot poorer than for MTPs and bioreactors. This was likely due to variations in morphology between flasks and problems with pellet formation and wall growth.

## Discussion

Discovery of new antibiotics and improvement of existing strains and processes are major goals for the fermentation industry. Efficient discovery and improvement programs require high-throughput cultivations platforms. The outcome of these screenings is highly dependent on high reproducibility. Furthermore, well-controlled conditions are essential to allow for extrapolation of the results to larger scales. The current study demonstrates the successful development of a deepwell MTP-based platform for cultivation of *S. coelicolor *and investigations of secondary metabolite production. Optimization of the inoculation procedure, the use of crushed, frozen mycelia, optimization of the buffer and addition of glass beads resulted in high reproducibility and that the same performance as in bench-scale reactors could be achieved (STDDEV: 5-10%). Hence, the 24-square deepwell MTP platform described here provides a reliable cultivation platform for understanding physiology and metabolic behavior. The MTP method that we developed has been successfully used by Siebenberg *et al. *(2010) and Dangel *et al. *(2010) [16,21] to investigate production of novobiocin. Moreover, the platform has also in our lab been effectively applied to study secondary metabolite production by other actinomycetes with only minor adjustments, e.g. glycopeptides production by *Amycolatopsis balhimycina *[unpublished data].

At the beginning of this work, before optimization, we observed significant discrepancy with respect to specific growth rate and volumetric production rate as compared to bioreactors and shake flasks, which is in agreement with the report by Minas *et al. *(2000) [22]. Well to well variation was another observation. This discrepancy was most likely a result of the pellety morphology in MTPs. This was addressed during the optimization procedure by inclusion of MOPS buffer and addition of glass beads, resulting in a dispersed, highly reproducible morphology and physiological behavior.

Classical methods of propagation include preparation of spore plates, harvest of spores and inoculation of spores into seed medium for germination. The resulting culture is then inoculated into production medium. This procedure might introduce variability at each step and is rather time consuming. An alternative could be to directly inoculate with spores in the production medium. However, spores are hydrophobic in nature and hence equal distribution in the culture medium may be problematic. Also, direct inoculation of spores into production medium may lead to longer lag phase, particularly if a minimal medium is used in this step. To circumvent these drawbacks, we applied frozen mycelia for direct inoculation, which we earlier successfully have applied to novobiocin production [16]. Both the classical method, with a pre-germination step, and the procedure with direct inoculation of spores require preparation of fresh spores and that takes approximately 8 days. A main advantage of using FM is that the time required to start the main cultivation is drastically reduced. In addition, the dispersed property enhances equal distribution of cells, making FM convenient to use for inoculation of miniaturized versions of cultivations and improves reproducibility.

Shake flasks have traditionally been the choice for screening studies as they allow for relatively small volumes and are easy to set up and clean compared to bench-scale reactors. The drawback compared to bench-scale is typically that conditions are less controlled, limitations in mass transfer may be an issue and the limitation in volume often results in that only end-point samples are taken. We observed high variations between shake flasks cultivations and reduced final concentrations and volumetric production rates for antibiotics. Our results are in agreement with those obtained by Siebenberg *et al. *(2010) [16] that also report lower final antibiotic concentrations and higher variation in data for shake flasks compared to MTPs. There are studies demonstrating superior oxygen transfer rates in deepwell MTPs compared to shake flasks [15] and we believe that this in addition to difficulties with controlling the morphology may be important underlying reasons for the poor performance of shake flasks as antibiotic production is known to be greatly influenced by oxygen levels and morphology.

## Conclusion

24-square deepwell plates with 3 ml working volume were used for *S. coelicolor *A3(2) cultivations. 6 beads (ø 3 mm) were included in each well and MOPS buffer was added to the medium to achieve dispersed and reproducible morphology. A comparative study was undertaken to verify agreement of extracellular parameters (specific growth rate, volumetric production rates for antibiotics) across different scales i.e. bioreactors (1 L), shake flasks (50 ml) and MTPs (3 ml). The MTP platform developed here performed highly reproducible and physiological data demonstrated good agreement with data from bioreactors. Shake flasks showed discrepancy with respect to significantly reduced volumetric production rates of antibiotics. Hence, MTPs are preferred over shake flasks for high-throughput physiology studies in filamentous microorganisms.

## Abbreviations

exp: Exponential; UDP: Undecylprodigiocin; ACT: Actinorhodin; μ_max_,: Maximal specific growth rate; MTPs: Microtiter plates; SFs: Shake flasks; DCW: Dry cell weight; FM: Frozen mycelia; q: Volumetric production rate; Y_sx_: Biomass yield on substrate; r_s_: Specific glucose consumption rate.

## Competing interests

The authors declare that they have no competing interests.

## Authors' contributions

SVS and PMB carried out the experimental work, performed data analysis and contributed to drafting of the manuscript. AEL initiated the study and contributed to experimental design, interpretation of data and manuscript writing. All authors read and approved the final manuscript.
